# Real-world clinical usage and efficacy of apalutamide in men with nonmetastatic castration-resistant prostate cancer: a multi-institutional study in the CsJUC

**DOI:** 10.1093/jjco/hyaf025

**Published:** 2025-02-02

**Authors:** Yoichiro Tohi, Keita Kobayashi, Kei Daizumoto, Yohei Sekino, Hideo Fukuhara, Heima Niigawa, Satoshi Katayama, Ryutaro Shimizu, Atsushi Takamoto, Kenichi Nishimura, Taichi Nagami, Yushi Hayashida, Hiromi Hirama, Koji Shiraishi, Ryotaro Tomida, Kohei Kobatake, Keiji Inoue, Yoshiyuki Miyaji, Kensuke Bekku, Shuichi Morizane, Noriyoshi Miura, Koichiro Wada, Mikio Sugimoto, Chu-shikoku Japan Urological Consortium, Keiji Inoue, Shinkuro Yamamoto, Koji Shiraishi, Yoshiyuki Miyaji, Nobuyuki Hinata, Junya Furukawa, Motoo Araki, Tatsushi Kawada, Atsushi Takenaka, Takashi Saika, Koichiro Wada, Yoichiro Tohi, Takuma Kato, Hirohito Naito, Hideo Fukuhara, Keita Kobayashi, Shin Ohira, Kei Daizumoto, Yutaro Sasaki, Ryotaro Tomida, Satoshi Katayama, Ryutaro Shimizu, Kenichi Nishimura, Ryuta Watanabe, Taichi Nagami, Atsushi Takamoto, Heima Niigawa, Yohei Sekino, Kohei Kobatake, Mikio Sugimoto

**Affiliations:** Department of Urology, Faculty of Medicine, Kagawa University, 1750-1 Ikenobe, Miki-cho, Kita-gun, Kagawa 761-0793, Japan; Department of Urology, Graduate School of Medicine, Yamaguchi University, 1-1-1, Minami-Kogushi, Ube, Yamaguchi 755-8505, Japan; Department of Urology, Tokushima University Graduate School of Biomedical Sciences, 3-18- 15 Kuramoto, Tokushima 770-8503, Japan; Department of Urology, Graduate School of Biomedical and Health Sciences, Hiroshima University, 1-2-3 Kasumi, Minami-ku, Hiroshima 734-8553, Japan; Department of Urology, Kochi Medical School, 185-1, Kohasu, Oko, Nankoku, Kochi 783-8505, Japan; Department of Urology, Kawasaki Medical School, 577, Matsushima, Kurashiki, Okayama 701-0192, Japan; Department of Urology, Okayama University Graduate School of Medicine, Dentistry and Pharmaceutical Sciences, 2-5-1 Shikata-Cho, Kita-Ku, Okayama 700-8558, Japan; Division of Urology, Department of Surgery, Faculty of Medicine, Tottori University, 86 Nishi-cho, Yonago, Tottori 683-8503, Japan; Department of Urology, Fukuyama City Hospital, 5-23-1, Zao, Fukuyama, Hiroshima 720-8505, Japan; Department of Urology, Ehime University, 454, Shitsukawa, Toon, Ehime 791-0295, Japan; Department of Urology, Shimane University Faculty of Medicine, Shimane, Japan; Department of Urology, Sakaide City Hospital, 3-2-1, Kotobuki, Sakaide, Kagawa 762-8550, Japan; Department of Urology, KKR Takamatsu Hospital, 4-18, Tenjin, Takamatsu, Kagawa 760-0018, Japan; Department of Urology, Graduate School of Medicine, Yamaguchi University, 1-1-1, Minami-Kogushi, Ube, Yamaguchi 755-8505, Japan; Department of Urology, Tokushima University Graduate School of Biomedical Sciences, 3-18- 15 Kuramoto, Tokushima 770-8503, Japan; Department of Urology, Graduate School of Biomedical and Health Sciences, Hiroshima University, 1-2-3 Kasumi, Minami-ku, Hiroshima 734-8553, Japan; Department of Urology, Kochi Medical School, 185-1, Kohasu, Oko, Nankoku, Kochi 783-8505, Japan; Department of Urology, Kawasaki Medical School, 577, Matsushima, Kurashiki, Okayama 701-0192, Japan; Department of Urology, Okayama University Graduate School of Medicine, Dentistry and Pharmaceutical Sciences, 2-5-1 Shikata-Cho, Kita-Ku, Okayama 700-8558, Japan; Division of Urology, Department of Surgery, Faculty of Medicine, Tottori University, 86 Nishi-cho, Yonago, Tottori 683-8503, Japan; Department of Urology, Ehime University, 454, Shitsukawa, Toon, Ehime 791-0295, Japan; Department of Urology, Shimane University Faculty of Medicine, Shimane, Japan; Department of Urology, Faculty of Medicine, Kagawa University, 1750-1 Ikenobe, Miki-cho, Kita-gun, Kagawa 761-0793, Japan

**Keywords:** apalutamide, nonmetastatic castration-resistant prostate cancer, prostate cancer, prostate-specific antigen response, PSA-doubling time

## Abstract

**Objective:**

To evaluate the real-world clinical usage and effectiveness of apalutamide in men with nonmetastatic castration-resistant prostate cancer (nmCRPC).

**Methods:**

We retrospectively reviewed the data of 186 men who received apalutamide across 17 institutions. The primary outcomes were the clinical usage of apalutamide for nmCRPC: prior usage of other androgen receptor signaling inhibitors (ARSIs), prior radical treatment, and the distribution of the prostate-specific antigen (PSA) doubling time (PSA-DT) at the initial administration of apalutamide. The secondary outcomes were the efficacy of apalutamide: PSA response (50% or 90% decline), progression-free survival, and skin-adverse events (AEs).

**Results:**

We identified 75 patients with nmCRPC. A total of 31 (41.3%) patients received prior treatment with other ARSIs. A total of 42 men (56%) did not receive any prior radical treatment. The PSA-DT was <3.0, 3.0–5.9, 6.0–10, and > 10 months in 34.7%, 40%, 14.7%, and 10.6% of the patients, respectively. Patients receiving prior treatment with other ARSIs showed a significantly lower PSA response (PSA 50% decline, 88.4% vs. 18.8%; PSA 90% decline, 60.5% vs. 6.2%, *P* < .001, respectively) and significantly shorter progression-free survival (median: 37 months vs. 4 months; log-rank *P* < .001) than those without prior ARSI treatment, although cancer status did not differ between the groups. Skin-AEs were observed in 42.7%.

**Conclusions:**

This real-world study revealed that apalutamide was used for the treatment after other ARSIs in >40% of patients with nmCRPC and showed limited efficacy in this context, although the effectiveness of apalutamide without prior other ARSI treatment was comparable with that reported in clinical trial results.

## Teaser

The effectiveness of apalutamide without prior ARSI treatment was comparable with previous clinical trials. However, in real world clinical practice, apalutamide was used after ARSI treatment in ~40% of nmCRPCs, with limited efficacy.

## Introduction

Nonmetastatic castration-resistant prostate cancer (nmCRPC) represents a clinically significant stage of prostate cancer (PC) characterized by rising prostate-specific antigen (PSA) levels despite castrate levels of testosterone and no radiographic evidence of metastasis. Effective management of nmCRPC is crucial because of the high risk of metastasis and the significant effects on metastatic CRPC (mCRPC) morbidity and mortality [1]. Treatment with androgen receptor signaling inhibitors (ARSIs) has been reported to delay mCRPC progression and improve mortality [2–5]. Apalutamide, an ARSI, has demonstrated efficacy in prolonging metastasis-free survival and overall survival (OS) in patients with nmCRPC in the phase III clinical trial SPARTAN [2,3].

Nevertheless, real-world data on the usage and efficacy of apalutamide outside the controlled conditions of clinical trials are limited in Japan [6,7]. In particular, real-world clinical data have shown that apalutamide is often associated with skin adverse events (AEs) in Japanese patients [8–10], although the mechanism underlying these AEs is not clearly understood [11], and concerns have been raised regarding its clinical usage in nmCRPC. In addition, the sequential treatment of nmCRPC with ARSIs represents an unmet medical need in patients showing increasing PSA levels even in the absence of metastasis on imaging. These findings highlight the need to evaluate the performance of apalutamide in real-world clinical practice, especially since patients may receive apalutamide as sequential therapy after failure of other ARSIs in nmCRPC. However, the limited clinical information on this topic hinders further understanding of this issue [12].

In the light of these gaps in knowledge, this study aimed to evaluate the real-world clinical usage and efficacy of apalutamide in patients with nmCRPC across multiple institutions in Japan.

## Patients and methods

### Ethics statements

This study was approved by the Institutional Review Board of Kagawa University (admission number: 2023-110). Kagawa University conducted a comprehensive ethics review of the other 17 facilities, which only provided existing samples and information. All procedures adhered to the ethical standards set by the relevant committees on human experimentation at the institutional and national levels and the principles outlined in the 1964 Helsinki Declaration and its subsequent revisions. The requirement for informed consent was waived due to the retrospective nature of the study. However, information about the study was made available on the website, and patients were allowed to decline participation if they wished.

### Patients

We retrospectively reviewed the data of patients treated with apalutamide across 17 Japanese institutions, mainly academic hospitals, in the Chu-shikoku region of Japan between May 2020 and April 2023.

### Data collection and outcome evaluation

Data for patient characteristics, including age at the initial administration of apalutamide, Eastern Cooperative Oncology Group Performance Status (Common Toxicity Criteria, Version 2.0 Publish Date 30 April 1999), weight, height, body mass index, PSA level at the initial administration of apalutamide, Gleason grade group at the initial diagnosis, clinical T stage at the initial diagnosis, prior use of ARSIs or chemotherapy, history of radical treatment, distribution of PSA-doubling time (PSA-DT) at the initial administration of apalutamide, and skin AEs, were retrospectively collected from the patient’s medical records. The full initial administration of apalutamide was set to 240 mg, and the reduced dose was set to 180 mg or less.

The primary outcome was real-world clinical usage: prior usage of other ARSIs, prior radical treatment, and the distribution of PSA-DT at the initial administration of apalutamide. PSA-DT was evaluated <3.0, 3.0–5.9, 6.0–10, and >10 months based on previous report [7]. The secondary outcomes were related to the efficacy of apalutamide and skin-AEs: PSA response (50% or 90% decline), progression-free survival (PFS; PSA level or radiographic), and OS. PSA response rates were determined by measuring the proportion of men who showed 50% or 90% decline in PSA values from baseline. Radiographic progression was defined by the presence of metastases on computed tomography or conventional bone scintigraphy scans. Time to disease progression was defined as the time from the initial administration of apalutamide to radiographic and PSA progression according to the Prostate Cancer Clinical Trials Working Group 2 criteria [13]. OS was defined as the time from the initial administration of apalutamide to death from any cause. We divided patients into two groups based on the use of other ARSIs prior to apalutamide (prior ARSI treatment and no prior ARSI treatment group) and compared PSA response, PFS, and OS between the groups. Regarding skin-AEs, the incidence of skin-AEs, time to the first incidence of skin AEs, the association between skin-AEs and PFS were examined.

### Statistical analysis

Quantitative variables were reported as medians with interquartile ranges (IQRs). Categorical variables were compared between the two groups using the chi-square and Fisher exact tests, whereas continuous variables were compared using the Mann–Whitney *U* test. Time-to-event analysis was performed using the Kaplan–Meier method. The results were presented as medians, 95% confidence intervals (CIs), and *P* values. The results of the Cox proportional hazards regression analyses were presented as hazard ratio (HR), 95% CIs, and *P* values. Statistical significance was defined as *P* < .05. All statistical analyses were performed using EZR (Saitama Medical Center, Jichi Medical University, Saitama, Japan), a graphical user interface for R (The R Foundation for Statistical Computing, Vienna, Austria) [14] and GraphPad Prism version 9.0.0 (GraphPad Software, San Diego, CA, USA).

## Results

### Patient characteristics

In the overall cohort of 186 PC patients treated with apalutamide, nmCRPC was identified in 75 men. Table 1 shows the overall characteristics of patients with nmCRPC. The median follow-up period from the initial administration of apalutamide to the last visit was 17.0 months (IQR, 9.0–32.5 months). The median patient age was 79 years (IQR, 72–84 years). Among the 75 patients, 42 (56%) had received no prior radical treatment, 10 (13.3%) had undergone surgery, and 23 (30.7%) had undergone radiation therapy.

**Table 1:** Patients’ characteristics

Variable	Total
Number, *n* (%)	75
Median age at initial dose, years (IQR)	79 (72–84)
Median height, cm (IQR)	161.1 (158.2–165.0)
Median weight, kg (IQR)	62.0 (54.4–70)
Median BMI, kg/m^2^, (IQR)	23.6 (21.4–26.3)
ECOG PS, n (%)	
0	53 (70.7)
1	17 (22.7)
2	5 (6.6)
Gleason grade group at initial diagnosis, n (%)	
1	6 (8.0)
2	7 (9.3)
3	5 (6.7)
4	14 (18.7)
5	37 (49.3)
Missing	6 (8.0)
T stage at initial diagnosis, n (%)	
T2	6 (8.0)
T3a	25 (33.3)
T3b	29 (38.7)
T4	10 (13.3)
Tx	5 (6.7)
History of prior radical treatment, n (%)	
None	42 (56)
Surgery	10 (13.3)
Radiation therapy	23 (30.7)
Prior usage of ARSI/Chemotherapy, n (%)	
ARSI	31 (41.3)
Chemotherapy	6 (8)
Types of prior ARSI	
Enzalutamide	13 (17.3)
Abiraterone	5 (6.7)
Darolutamide	2 (2.7)
Enzalutamide and Abiraterone	11 (14.7)
Median PSA at initial administration, ng/ml	3.04 (1.25–6.74)
Median PSA-DT, month (IQR)	3.60 (2.30–5.65)
Initial dose of apalutamide, n (%)	
240 mg	57 (76)
180 mg	3 (4)
120 mg	15 (20)
Median follow-up period (IQR)	17.0 (9.0–32.5)

BMI, body mass index; ECOG PS, Eastern Cooperative Oncology Group Performance Status

### Real-world clinical usage of apalutamide

**Figure 1:**
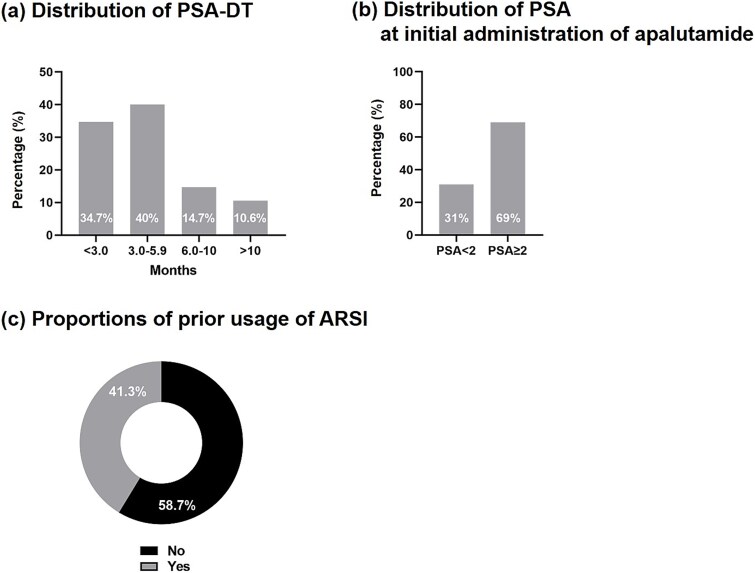
(a) Distribution of PSA-DT values, (b) distribution of PSA levels at the initial administration of apalutamide, and (c) proportions of patients who had previously received ARSIs.

PSA-DT was <3.0 months in 34.7% (*n* = 26), and 3.0–5.9 months in 40% (*n* = 30), 6.0–10 months in 14.7% (*n* = 11), and > 10 months in 10.6% (*n* = 8) of the patients (Fig. 1a). The distribution of PSA levels at the initial administration of apalutamide was PSA ≥ 2 in 69% of the patients and PSA <2 in 31% (Fig. 1b). A total of 31 patients (41.3%) received prior treatment with other ARSIs (Fig. 1c) (Supplementary Fig. 1). All six patients who used chemotherapy were treated with other ARSI (Table 1). Regarding prior ARSI treatment, patients treated with two or more ARSIs included 14.7% (*n* = 11) of the patients (Table 1). The initial administration of apalutamide was 240 mg in 57 men (76%), 180 mg in three men (4.0%), and 120 mg in 15 men (20%) (Table 1).

### Efficacy of apalutamide

**Figure 2:**
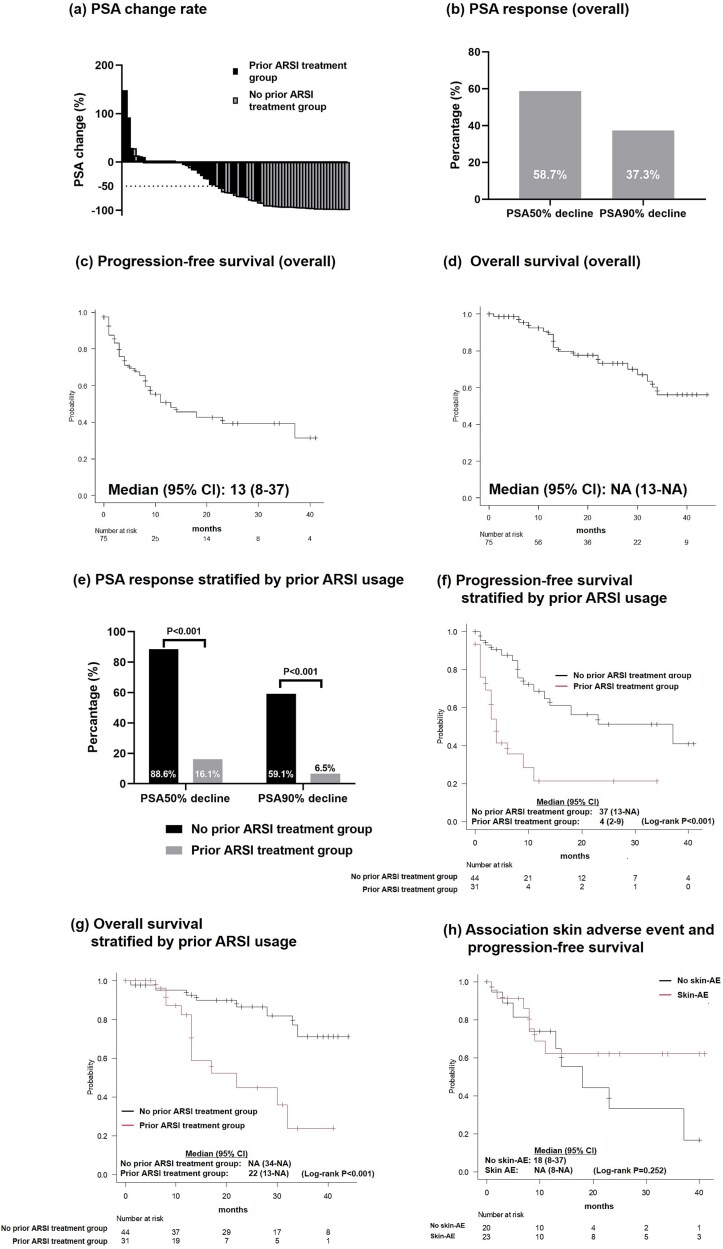
(a) PSA change rate, (b) PSA response in the overall cohort, (c) PFS in the overall cohort, (d) OS in the overall cohort, (e) PSA response stratified by prior ARSI usage, (f) PFS stratified by prior ARSI usage, (g) OS stratified by prior ARSI usage, and (h) association of skin AEs with PFS.

In the overall cohort, the maximal percentage change from baseline in PSA levels is shown in Fig. 2a. The rates of patients achieving PSA 50% and PSA 90% declines during the study period were 58.7% and 37.3%, respectively (Fig. 2b). Both the median PFS and OS calculated using the Kaplan–Meier method were 13 months (95% CI, 8–37 months) and not reached [95% CI, 13 months-not available (NA)] (Fig. 2c and d). During the study period, disease progression was observed in 35 men (46.7%) and PC-related deaths occurred in 11 men (14.7%).

Next, we compared the efficacy of apalutamide between prior ARSI treatment and no prior ARSI treatment group. The median PSA level at the initial administration of apalutamide, PSA-DT, and proportion of patients who received the initial full dose of apalutamide did not differ between the groups (Supplementary Table 1). The median follow-up period differed between the groups (23.0 months vs. 12.0 months, *P* = .001) (Table 2). In prior ARSI treatment group, the PSA response was significantly lower than that in no prior ARSI treatment group (PSA 50% decline, 88.6% vs. 16.1%; PSA 90% decline, 59.1% vs. 6.5%, *P* < .001, respectively; Fig. 2e). The PFS in prior ARSI treatment group was significantly shorter [median, 37 months (95% CI, 13 months-NA) vs. 4 months (95% CI, 2–9 months), log-rank *P* < .001] than that in no prior ARSI treatment group (Fig. 2f). In addition, the OS in prior ARSI treatment group was significantly shorter [median NA (95% CI, 34 months-NA) vs. 22 months (95% CI, 13 months-NA), log-rank *P* < .001] than that in no prior ARSI treatment group (Fig. 2g). After adjusting for age, PSA-DT, prior ARSI treatment, and Gleason grade group>3, multivariable Cox proportional hazards regression analyses revealed that prior ARSI treatment was significantly associated with PFS (HR 5.273, 95% CI 2.355–11.81, *P* < .001) (Table 2).

### Skin AEs during apalutamide treatment

Of the 75 men treated with apalutamide, 57.3% (*n* = 43) experienced AEs of any grade, while 12% (*n* = 9) experienced grade 3 or higher AEs. Regarding any-grade AEs, skin-related AEs, fatigue, liver dysfunction, loss of appetite, hypertension, myocardial infarction, hearing loss, and arthritis were observed in 42.7% (*n* = 32), 5.3% (*n* = 4), 2.7% (*n* = 2), 2.7% (*n* = 2), 1.3% (*n* = 1), 1.3% (*n* = 1), 1.3% (*n* = 1), and 1.3% (*n* = 1) of the patients, respectively. Among grade 3 or higher AEs, grade 3 skin-related AEs occurred in 10.7% (*n* = 8) of the patients, and myocardial infarction was observed in 1.3% (*n* = 1). The median time to the first incidence of skin AEs was 61.5 days (IQR, 53–84 days). In the cohort receiving first-line apalutamide therapy, PFS was not significantly different between the group showing skin AEs and the group without skin AE (*P* = .72; Fig. 2h).

## Discussion

Using real-world data from multiple institutions, we examined the clinical usage of apalutamide and clinical outcomes such as PSA response, PFS, and the incidence of skin AEs during apalutamide treatment in Japanese men with nmCRPC. This study highlights several critical aspects of apalutamide usage in real-world clinical practice for men with nmCRPC. First, our findings indicated that a substantial proportion of men received prior treatment with other ARSIs (41.3%), reflecting the complex treatment landscape in the nmCRPC population. This indicates that this population represents cases of nmCRPC with elevated PSA levels in the absence of metastases on imaging. Notably, the PFS and OS in these patients receiving prior treatment with other ARSIs were significantly shorter than those without prior ARSI treatment (Fig. 2f and g). This was evident from the markedly reduced PSA response rates in men treated with apalutamide after treatment with other ARSIs (Fig. 2e). The limited efficacy of apalutamide in settings of receiving prior treatment with other ARSIs underscores the challenges in managing nmCRPC with sequential ARSI therapy. To elucidate the treatment strategy for nmCRPC, further studies are needed to evaluate metastasis-free survival in patients who continue to receive the first-line ARSI until metastasis versus those who receive sequential ARSI therapy in the context of PSA progression. Because of the well-known cross-resistance between ARSI agents in patients showing mCRPC [15], the use of sequential ARSI therapy in nmCRPC should be avoided as much as possible, and docetaxel and cabazitaxel should be promptly administered in cases where chemotherapy is appropriate.

In contrast to the findings obtained for sequential ARSI therapy, apalutamide treatment without prior ARSI treatment was shown to be effective in this study. The median PFS of 37 months with apalutamide treatment without prior ARSI treatment (Fig. 2f) is similar to the results of the SPARTAN trial, although the endpoint was different because of metastasis-free survival [2,3]. In addition, this study showed that PSA 50% and PSA 90% declines were observed in 88.4% and 60.5% of patients receiving apalutamide treatment without prior ARSI treatment, respectively (Fig. 2e). These results were comparable with those of the SPARTAN subgroup analyses [16]. In real-world clinical practice, apalutamide for nmCRPC shows potential for achieving a deep PSA response. Our study suggests that while apalutamide is effective when used as treatment without prior ARSI, its benefits diminish when used after prior other ARSI treatments.

This study highlights a notable difference in the use of apalutamide in real-world clinical practice in comparison with clinical trials. Specifically, while the SPARTAN trial focused on patients with PSA-DT of 10 months or less [2,3], indicating relatively rapid disease progression, our cohort included a broader range of PSA-DT values. Thus, PSA-DT values >10 months were observed in 10.6% of the cases (Fig. 1a). Additionally, ~30% of the men in this study had PSA levels <2 ng/ml when they received the initial administration of apalutamide (Fig. 1b), which contrasts with the clinical guideline definitions of CRPC [17]. These findings suggest that in real-world clinical practice, clinicians apply apalutamide in a wider variety of clinical scenarios than those strictly defined in clinical trials. This real-world application of apalutamide reflects clinicians’ efforts to adapt trial results to the nuanced and diverse situations encountered in clinical practice within the bounds of the approved indications of the drug. Although the application of clinical trial outcomes to real-world clinical practice is not straightforward because of factors such as restrictive enrollment criteria, biological variability, publication bias, and limitations in experimental design [18], the limited efficacy of ARSI sequential therapy observed in this study further underscores the importance of real-world data analysis and provides crucial insights that can inform clinical decision-making.

**Table 2:** Multivariable Cox proportional hazards regression analysis for PFS

	Hazard ratio	95% CI	*P* value
Age	1.018	0.965–1.074	0.514
PSA-DT	1.05	0.950–1.161	0.337
Prior ARSI usage	5.273	2.355–11.81	<0.001
Gleason grade group >3	1.022	0.441–2.368	0.959

The incidence of skin AEs after apalutamide treatment for nmCRPC in this study was almost comparable with the results of the integrated analysis of clinical trials [11] and previous reports based on real-world clinical practice [8–10]. In this study, the incidence of skin AEs was 42.7%, and the median time to first incidence was ~2 months. To assess whether skin AEs were associated with treatment response, we compared PFS in the presence or absence of skin AEs and found no significant differences between groups, consistent with the findings of previous studies on apalutamide for metastatic castration-sensitive PC [8,19]. However, further analyses with greater statistical power are required.

Despite its multi-institutional nature, this study had some limitations. First, the study had a selection bias. Owing to the multi-institutional retrospective nature of the study, the selection criteria for apalutamide, initiating or reducing the apalutamide dose were inconsistent. The attending physicians determined the timing of apalutamide administration based on the patient’s disease status. In this context, this study, which used real-world clinical data, had a different background from that of clinical trials. Additionally, AE data collection was limited by the retrospective nature of the study. Second, we did not perform multivariate analysis because of the limited number of mortality-related events. Therefore, additional studies with larger sample sizes are required to validate our findings. Third, this study did not evaluate the efficacy of apalutamide compared with other ARSIs or taxanes in the nmCRPC setting. While our findings demonstrated that patients with prior treatment using other ARSIs exhibited worse PSA decline and shorter PFS than those without prior ARSI treatment, it is crucial to acknowledge that this study did not compare the efficacy of apalutamide with other ARSIs or taxanes following ARSI treatment failure. Therefore, future studies are needed to directly compare apalutamide with other ARSIs or taxanes in this clinical context to better understand the effectiveness of sequential ARSI treatment. These comparisons are essential to guide optimal treatment sequencing and improve clinical outcomes in nmCRPC management. Fourth, due to the retrospective study design, information on the exact duration of prior ARSI treatment was lacking, which prevented us from assessing the efficacy of sequencing to apalutamide based on the type and duration of prior treatment responses. However, cross-resistance among ARSI drugs in mCRPC patients is well known [15]. The implication of this study is that understanding the real-world applications, efficacy, and safety of apalutamide in diverse patient populations can provide valuable insights for optimizing treatment strategies and addressing unmet clinical needs.

In conclusion, while apalutamide has been shown to be effective in Japanese men with nmCRPC, this real-world study revealed that apalutamide was used for sequential ARSI treatment in >40% of the patients, and its efficacy in sequential treatment regimens was poorer than that in first-line setting.

## Supplementary Material

Supplementary_fig_r1_hyaf025

Supplementary_table_r1_hyaf025

## Data Availability

The datasets generated and analyzed during this study are available from the corresponding author upon reasonable request.
