# Supplementary figures and images for: Real-world clinical usage and efficacy of apalutamide in men with nonmetastatic castration-resistant prostate cancer: a multi-institutional study in the CsJUC
Source: Jpn J Clin Oncol. 2025 Feb 2;55(6):643–9. doi: 10.1093/jjco/hyaf025 (PMC12138768; doi:10.1093/jjco/hyaf025)

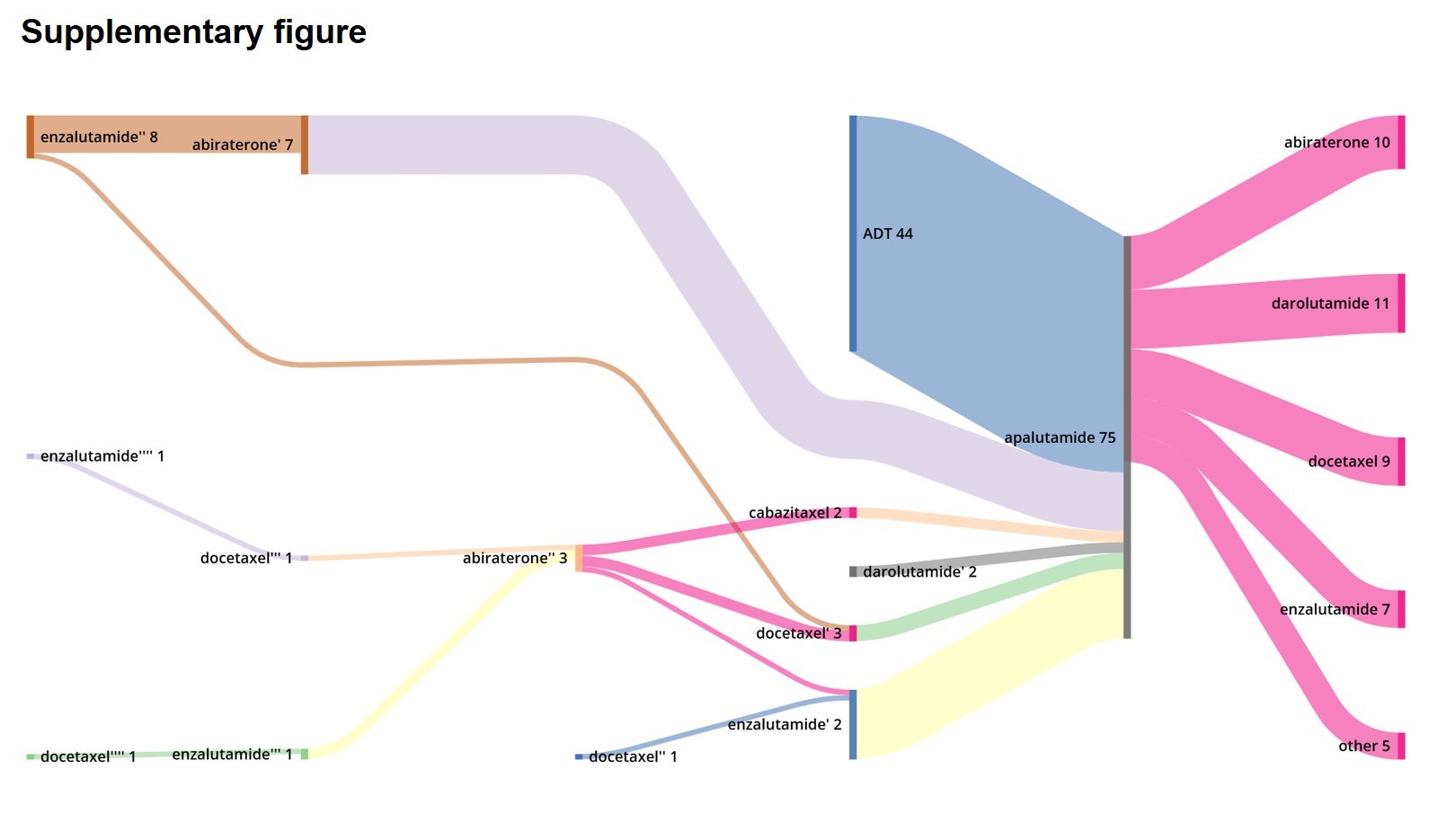

Supplement: Supplementary_fig_r1_hyaf025 [file supplementary_fig_r1_hyaf025.jpeg]
