# Supplementary material for: Real-world clinical usage and efficacy of apalutamide in men with nonmetastatic castration-resistant prostate cancer: a multi-institutional study in the CsJUC
Source: Jpn J Clin Oncol. 2025 Feb 2;55(6):643–9. doi: 10.1093/jjco/hyaf025 (PMC12138768; doi:10.1093/jjco/hyaf025)
Supplement: Supplementary_table_r1_hyaf025 [file supplementary_table_r1_hyaf025.docx]

Supplementary table Patients' characteristics stratified by prior usage of other ARSIs

| Variable | No prior ARSI treatment group | Prior ARSI treatment group | P value |
| --- | --- | --- | --- |
| Number, n (%) | 44 | 31 |  |
| Median age at initial dose, years (IQR) | 81 (72-85) | 78 (73.5-83.5) | 0.983 |
| Median BMI, kg/m^2^, (IQR) | 23.6 (21.4-26.4) | 23.6 (21.4-25.6) | 0.638 |
| ECOG PS 0-1, n (%) | 40 (90.9) | 30 (96.8) | 0.397 |
| Gleason grade group >3 at initial diagnosis, n (%) | 32 (78) | 24 (85.7) | 0.779 |
| T stage ≥3 at initial diagnosis, n (%) | 36 (87.8) | 28 (96.6) | 0.389 |
| No prior radical treatment, n (%) | 17 (38.6) | 16 (51.6) | 0.346 |
| Median PSA at initial dose, ng/mL | 2.81 (1.03-4.48) | 3.9 (1.88-7.72) | 0.188 |
| Median PSA-DT, month (IQR) | 3.70 (2.28-6.08) | 3.60 (2.25-5.10) | 0.842 |
| Initial full dose of apalutamide, n (%) | 36 (81.8) | 21 (67.7) | 0.180 |
| Median follow-up period (IQR) | 23.0 (13.8-36.5) | 12.0 (6.5-17.0) | 0.001 |

ARSI, androgen receptor signaling inhibitor; IQR, interquartile range; BMI, body mass index; ECOG PS, Eastern Cooperative Oncology Group Performance Status; PSA, prostate-specific antigen; PSA-DT, prostate-specific antigen doubling time
